# Genetic Analysis and Fine Mapping of the Fire Blight Resistance Locus of *Malus ×arnoldiana* on Linkage Group 12 Reveal First Candidate Genes

**DOI:** 10.3389/fpls.2021.667133

**Published:** 2021-04-20

**Authors:** Ofere Francis Emeriewen, Klaus Richter, Henryk Flachowsky, Mickael Malnoy, Andreas Peil

**Affiliations:** ^1^Julius Kühn Institute (JKI), Federal Research Centre for Cultivated Plants, Institute for Breeding Research on Fruit Crops, Dresden, Germany; ^2^Julius Kühn Institute (JKI), Federal Research Centre for Cultivated Plants, Institute for Resistance Research and Stress Tolerance, Quedlinburg, Germany; ^3^Research and Innovation Centre, Fondazione Edmund Mach (FEM), San Michele all’Adige, Italy

**Keywords:** *Erwinia amylovora*, *FB_Mar12*, MAL0004, recombinant individuals, region-of-interest

## Abstract

*Malus* ×*arnoldiana* accession MAL0004 has been found to be resistant to moderately and highly virulent strains of the fire blight causal pathogen – the Gram-negative bacterium, *Erwinia amylovora*. Genetic analyses with an F1 segregating population derived from crossing the highly susceptible apple cultivar ‘Idared’ and MAL0004 led to the detection and mapping of the fire blight resistance locus of *M. ×arnoldiana* to linkage group (LG)12 (*FB_Mar12*). *FB_Mar12* mapped at the distal end of LG12 below the apple SSR Hi07f01 in an interval of approximately 6 cM (Centimorgan), where both the fire blight resistance loci of *M. floribunda* 821 and ‘Evereste’ were located. We fine mapped the region containing *FB_Mar12* using 892 progenies. Mining of the region of interest (ROI) on the ‘Golden Delicious’ doubled haploid genome (GDDH13) identified the presence of 2.3 Mb (megabases) in the homologous region. Of 40 primer pairs designed within this region, 20 were polymorphic and nine were mapped, leading to the identification of 24 significant recombinant individuals whose phenotypes were informative in determining the precise position of the locus within a 0.57 cM interval. Analyses of tightly linked marker sequences on the *M. baccata* draft genome revealed scaffolds of interest putatively harboring the resistance loci of *M. ×arnoldiana*, a hybrid between *M. baccata* and *M. floribunda*. Open reading frame (ORF) analyses led to the prediction of first fire blight resistance candidate genes with serine/threonine kinase and leucine-rich repeat domains, including homologs of previously identified ‘Evereste’ candidate genes. We discuss the implications of these results on breeding for resistance to fire blight.

## Introduction

*Erwinia amylovora* (Burrill; Winslow et al., 1920) is the causative pathogen of fire blight – the most destructive bacterial disease of the domesticated apple (*Malus domestica* Borkh.). The Gram-negative bacterium uses the type III secretion system (T3SS) to deposit effector proteins into host cells thereby causing fire blight in susceptible hosts (Oh and Beer, 2005; Khan et al., 2012; Malnoy et al., 2012; Emeriewen et al., 2019; Yuan et al., 2020). Fire blight symptoms, such as blossom blight, necrosis, shepherd’s crook, shoot and rootstock blights, begin to develop the following entry of the pathogen *via* flowers and wounds into host plants and the subsequent internal spread within the host (Peil et al., 2009). Since most commercial apple cultivars are susceptible to fire blight, there is a heavy dependence on antibiotics’ application especially in the United States to avoid fire blight epidemics (McManus, 2014; Beckerman and Sundin, 2016) even until recently – in organic apple orchards (Johnson and Temple, 2013). Many European countries in contrast, prohibit the use of antibiotics, emphasize other control strategies, such as the application of antagonists or phytosanitary measures like pruning of infected tissues, and complete destruction of affected trees. Breeding of elite fire blight resistant cultivars (Peil et al., 2020) remains the silver bullet.

Although there is an abundance of genetic studies, which describe genomic regions that contribute to fire blight resistance in the genus *Malus* (reviewed in Emeriewen et al., 2019), several intertwined factors affect breeding for resistance to this devastating disease. Firstly, most cultivars with acceptable marketable characteristics are more or less susceptible to the pathogen. Nonetheless, fire blight quantitative trait locus (QTL) studies have led to the discovery of QTLs in apple cultivars explaining only less than 50% of the phenotypic variance (Calenge et al., 2005; LeRoux et al., 2010; Khan et al., 2013; Desnoues et al., 2018; van de Weg et al., 2018). Secondly, stronger resistance to fire blight is found in some wild apple accessions, which are the donors of mapped resistance QTLs, notably on linkage group (LG) 3 of *Malus ×robusta* 5 (Peil et al., 2007, 2019), on LG10 of *M. fusca* (Emeriewen et al., 2014a,b, 2020) and on LG12 of ‘Evereste,’ *M. floribunda* 821 (Durel et al., 2009) and *M. ×arnoldiana* (Emeriewen et al., 2017a). These wild *Malus* accessions have small and astringent fruits (Peil et al., 2020). Thirdly, resistance to fire blight is relative; it is highly dependent on the strain of the bacterium (Norelli and Aldwinckle, 1986; Peil et al., 2011; Vogt et al., 2013; Wöhner et al., 2014, 2018; Emeriewen et al., 2019).

The development of an elite cultivar that is resistant to fire blight is a key goal of resistance breeding. However, the introgression of the QTLs from apple cultivars, especially the QTL of ‘Fiesta’ on LG7 (Calenge et al., 2005), which is easier, faster, and more promising since it is from an elite background, is risky as the locus alone is not sufficient to provide reliable resistance. The use of resistance from wild species is especially difficult due to the several pseudo-backcrosses that is required to eliminate linkage drag – a situation that makes apple breeding in general to last for several decades, coupled with the long juvenile phase of the apple plant (Hanke et al., 2007, 2020; Gessler and Pertot, 2012). Nevertheless, the development of pre-breeding materials from wild apple accessions that are highly resistant to fire blight is crucial.

The long generation cycle of apple can be shortened by “Fast track” breeding (Volz et al., 2009; Baumgartner et al., 2014) or the “rapid cycle” breeding approach, using intermediate transgenic steps (Flachowsky et al., 2011; Le Roux et al., 2012; Schlathölter et al., 2018). The use of biotechnological approaches like the over expression of fire blight resistance genes (Broggini et al., 2014; Kost et al., 2015) or the knockdown of susceptibility genes (Campa et al., 2019; Pompili et al., 2020; Tegtmeier et al., 2020) could improve high quality cultivars in a reasonable time but one has to keep in mind that these trans- or cis-genetic techniques are not accepted by the general public at least in Europe.

Further, even a strong resistance can succumb to very virulent strains of *E. amylovora*. For example, the resistance of *M. ×robusta* 5 (Mr5) and the strong QTL on LG3 (Peil et al., 2007) is overcome by the very virulent Canadian strain Ea3049 (Peil et al., 2011; Vogt et al., 2013). Bacterial effector proteins play crucial roles in strain specificity. The breakdown of Mr5 resistance is the result of a single mutation in the *avrRpt2_EA_* effector of *E. amylovora* or the deletion of this effector (Vogt et al., 2013; Emeriewen et al., 2019). Similarly, the deletion of the *eop1* effector of *E. amylovora* has been implicated in the potential breakdown of the resistances of *M. floribunda* 821 (Mf821) and ‘Evereste’ (Wöhner et al., 2018; Emeriewen et al., 2019) but not of MAL0004. Thus, durable resistance to fire blight requires the pyramiding of several genes/QTLs, which potentially possess different resistance mechanisms. Thus far, in the genus *Malus*, only a single gene for fire blight resistance, *FB_MR5* (Fahrentrapp et al., 2013), underlying the Mr5 fire blight resistance QTL on LG3 (Peil et al., 2008), is found to be functional (Broggini et al., 2014). However, this gene has been overcome by *E. amylovora* strains, thereby highlighting the importance of identifying alternative and novel genes and their functional alleles (and linked molecular markers), which would be useful for pyramiding to obtain durable resistance to fire blight and for marker-assisted selection of breeding materials.

There are a few fire blight resistance candidate genes with potential novelty. A receptor-like kinase gene (*FB_Mfu10*) was proposed for the major fire blight resistance QTL region of *M. fusca* (Emeriewen et al., 2018). The donor of this gene, *M. fusca* accession MAL0045, is highly resistant to strains that break the resistance of Mr5; and its corresponding QTL on LG10 is not broken down by these strains (Emeriewen et al., 2015, 2017b), suggesting that the underlying gene acts in a different manner as *FB_MR5*. However, a functional proof of *FB_Mfu10* in complementing studies is still required. Furthermore, Parravicini et al. (2011) proposed receptor-like kinase and NBS-LRR candidate genes in the fire blight QTL region of the ornamental cultivar ‘Evereste’ on LG12 (Durel et al., 2009), but their functional proof is yet to be reported.

The basis of the present study is the fire blight resistance locus (*FB_Mar12*) of *M. ×arnoldiana* located on LG12 (Emeriewen et al., 2017a) in a similar region, where the fire blight QTLs of ‘Evereste’ (E12) and Mf821 (Mf12) are located (Durel et al., 2009). It is unclear if these three QTLs at the distal end of LG12 are distinct or distinct alleles of the same QTL/gene. However, while Mr5 resistance breaking-strain Ea3049 does not overcome *FB_Mar12* and only minimally affects its donor, *M. ×arnoldiana* – accession MAL0004 (Emeriewen et al., 2017a; Wöhner et al., 2018), there is no data confirming if E12 and Mf12 (Durel et al., 2009) are broken down by this strain or similar highly virulent strains. Results of inoculation experiments on all three donors of the resistance QTLs on LG12 with an *eop1* effector deletion mutant strain of *E. amylovora* showed that fire blight was induced in ‘Evereste’ and Mf821, but not in MAL0004 (Wöhner et al., 2018), indicating a possible difference in mechanisms. Thus, the strong resistance of MAL0004 to Ea3049 makes *FB_Mar12* an interesting candidate for durable resistance. Hence, identifying the gene underlying *FB_Mar12* is crucial.

Here, we fine mapped *FB_Mar12* region on LG12 and delimited the resistance region from 6.22 (Emeriewen et al., 2017a) to 0.57 cM by significantly increasing the number of mapping individuals and saturating the region with newly developed molecular markers. This approach facilitated the identification of recombinant individuals within the region of interest (ROI). Analyses of the homologous region in the draft reference genome of *Malus baccata* (Chen et al., 2019) led to the prediction of the first candidate resistance genes.

## Materials and Methods

### Plant Materials and DNA Extraction

*FB_Mar12* was initially detected using 145 individuals (07240 population) that constituted the progeny of a cross between ‘Idared’ and MAL0004 (Emeriewen et al., 2017a). To increase the mapping individuals, we performed two more crosses thus: ‘Idared’ × MAL0004, which resulted in 359 individuals, designated as 17229 population and ‘Golden Delicious’ × 07240–37 (a resistant progeny of the original 07240 population) that resulted in 388 individuals designated as 17230 population. In total, 892 individuals were used for this study (Table 1). DNA was first extracted from the 17229 and 17230 individuals by adding a piece of leaf (4 mm diameter) into 50 μl of extraction solution (Sigma Aldrich, Hamburg, Germany) followed by incubating in a thermocycler for 10 min at 95°C and then adding 50 μl of Extract-N-Amp plant dilution solution (Sigma Aldrich, Hamburg, Germany) before removing the leaf material. DNA was diluted 1:5 and stored in −20°C until required for PCR. After identifying interesting recombinant individuals following genotyping with markers, Qiagen DNeasy Plant Mini Kit (Qiagen, Hilden, Germany) was used to isolate DNA of these individuals, which formed the basis of subsequent analyses.

**Table 1:** Three populations used in this study with a total number of 892 progeny individuals.

Population name	Cross	Number of individuals	Number of recombinants in the interval from	
			FRMb251 to FRMb199	FRMb31M27a to FRMb197
^*^07240	‘Idared’ × MAL0004	145	13	7
17229	‘Idared’ × MAL0004	359	35	14
17230	07240–37 × GD	388	14	8
Total		892	62	29

* Original mapping population from Emeriewen et al. (2017a).

GD = ‘Golden Delicious.’

### Identification of Recombinant Individuals Within the Region of Interest

The *FB_Mar12* ROI was the interval between the markers FRMb251 and FRMb199, a 6.22 cM interval bracketing the QTL (Emeriewen et al., 2017a). FRMb251, FRMb199, and three additional markers in between (FRMb103x, CHFBE01, and CHFBE02) were used to genotype the 17229 and 17230 individuals in order to identify individuals that showed recombination events between FRMb251 and FRMb199. PCR for marker genotyping was performed in a multiplex using the Type-It kit (Qiagen, Hilden, Germany) according to the manufacturer’s protocol but in a 10 μl volume with the following conditions: 95°C for 5 min, followed by 30 cycles of 95°C for 1 min, 60°C for 1 min 30 s, and 72°C for 30 s, and a final extension for 30 min at 60°C. PCR fragments were analyzed on an ABI 3500xL Genetic Analyzer (Applied Biosystems, ThermoFisher Scientific, Darmstadt, Germany). The PCR products were diluted 1:100 and 1 μl of the dilution was mixed with 8.95 μl of HiDi formamide (Applied Biosystems) and 0.05 μl of Liz 600 size standard (Applied Biosystems) in a total volume of 10 μl. The mixture was denatured in a thermocycler at 94°C for 5 min before loading onto the ABI. The SSR fragments were analyzed using GeneMapper™ software version 6 (ThermoFisher Scientific, Darmstadt, Germany). Individuals, which showed recombination events, were used for further analyses. The entire 07240 individuals and identified recombinant individuals are maintained in greenhouses, nurseries, and in our orchard.

### Development of Closely Linked Markers and Polymorphism Tests

The sequences of FRMb251 and FRMb199 markers were BLAST-searched against the ‘Golden Delicious’ doubled haploid genome, GDDH13 (Daccord et al., 2017) to identify the corresponding ROI. This corresponded to the region between 30,406,247 and 32,679,162 (in megabases) in the GDDH13 genome sequences. Within the GDDH13 corresponding region, 40 primer pairs flanking SSR motifs were designed (Table 2). We used Primer3 program to design primers (Rozen and Skaletsky, 2000). The M13 forward primer (5'-TGTAAAACGACGGCCAGT-3') was attached to the forward primers designed from GDDH13 as described by Schuelke (2000) to economize costs and visualize the alleles on the ABI 3500xL Genetic Analyzer (Applied Biosystems, ThermoFisher Scientific, Darmstadt, Germany).

**Table 2:** Forty primer pairs developed from the ‘Golden Delicious’ doubled haploid genome in the region from 31,092,690 to 32,362,417.

Primer name	Forward sequence	Reverse sequence
FRMb31M22	TGGATTGAAAATTTGGGTGTC	GCTGGTGCATTTCCATTTTT
FRMb31M23	GACACCAAAACGAGCCCTTA	TCCATTTTTGGATTCTGAAGTTG
FRMb31M27a	AATGTGTGGTTCCTCCCAAA	CACGAGTTAACATTACCTTTGATTG
FRMb31M27b	TCTGAAGAGGAGATCAGAGAACC	GCCCAATTTCGGTTACACAT
FRMb31M30	CCAACTTTAAGGATCCAAATCG	CACAGCAGGTCGGACATTT
FRMb31M31	AACAGGTGCTTGAATAGTTGACA	CCAGCAATTACTGAAAGAGATCA
FRMb31M31a	AGCGCGTGGGACTATAGAAA	GGCTTTCTGAATTGCTTTGC
FRMb31M31b	GAGTGTTCAGAGCCCAAAGC	TTGGTGAAATGGGTGTCAGA
FRMb31M33	GTGGTTCCGGAATTGAGAGA	AGAATCAGAAGCCCTTCACG
FRMb31M35	GCCATTGTGATGATCTGATTTT	GCTGAGGGATGAGTTTTTGC
FRMb31M36a	ATGCATTTCATGGTTGCATA	TCACAATCACCCTAGATGCAG
FRMb31M36b	TCTTGGCATGTCATACATTTGAG	AAACGTGAGTGGGCAAAAAC
FRMb31M48	TTGCCTCTTACCCCCTTACC	AGATCGGGGAGAAGGAGAAA
FRMb31M52	AATTCCCAAGTTGCTGCTTC	CGGAATTCTGTTATTGCATTTTT
FRMb31M54	CCCTAACCAATCGAATCGAAA	CTCCCGGAATTCATCAAGAC
FRMb31M57	TCAAGACCAAGGCTTCACCT	GGCCCAAAGAAGAGAAGGTC
FRMb31M61a	GGTGGGGTTGAAAGATGAGA	AAGCAGCATCACTGGTGAGA
FRMb31M61b	GGGAGGAGGTTTGTCCCTTA	TGATGATGGTCCTTGTTGGA
FRMb31M67	CGCCTTGCCTAGACTCGTAT	CCGACTTAATCGTCAGCACA
FRMb31M69	AAGGAGCTAGCTTGCACTGG	GAGAGGGAAGGGATTTCACA
FRMb31M7	CAGACTCTGCAACCCCTCTC	AGTCAAGTGCTGCTGCAAGA
FRMb31M80	CTGGCCGTAGATGATCCAGT	GTTTGGCGAGAGAGAAAACG
FRMb31M82	TAGGCTCTGGTGCCAAGAAG	CAGATCACGGAACCCTAGAGA
FRMb31M84a	GGGCCAATGTCACACTAACA	ACGAACACGACAGACACGAC
FRMb31M84b	TGCACATCCATATTCCATCA	GTGGGTAACACGAAACACGA
FRMb31M85	CTGGCCGTAGATGATCCAGT	GTTTGGCGAGAGAGAAAACG
FRMb31M87	AAAGAGCTTTGCTTGGCTTG	TCTCAACTTTCCCACCAACC
FRMb32M039	CTCAGCCTGCTAGAGGAGCTA	CAGGCAAGTCTGATATTCTTGG
FRMb32M03x_F1^*^	TCTCAGCTTTTGAAGCACCA	CACCTTTGTTCCATCCGTCT
FRMb32M03x_F2^*^	TGAACGCCGATAGAGATTGA	
FRMb32M04a	GCTTTGGATTCCAGTTTAGATAAG	TTTGCTTGTATTTTCCATGACTG
FRMb32M04b	TGGACAAATTCAGTGACACCA	CAAACCACCCCAAATTCTGT
FRMb32M11	AAACATGAACCGGTTTGTCC	GCTGGCGGATGGATAGATTA
FRMb32M12	TGTCAATGATTTGTCCCCATA	TTGCAGCAAGGCTCATAAAA
FRMb32M16	GAGCCCCATGAACCTCAGTA	CCTGGCCATGCAATCTATCT
FRMb32M19	GTCGGCCATTCCTAAAACAA	AGAGTGGTGCATCATTGCAG
FRMb32M21	TTCTTGCATAATTTGCTCTGTGA	CAGTGGAGGAAAGGCAACAT
FRMb32M27	TTTAATTGGCTTTTCATTCACG	AAGGCGACTCATGATTTCGTA
FRMb32M33x	CCTTTGTTGGAAGGAGCTACA	CCATCTTACTCATTGTCGCTTA
FRMb32M33y	ATGCCCATCATCTTCTCCAC	TGGTAGAGCGTGGTATGGAA

* *Both forward primers had the same reverse primer*.

To test for polymorphism, developed primers were analyzed on ‘Idared,’ MAL0004, 07240-37 and ‘Golden Delicious’ and a subset of six progenies of the 07240 population, three of which inherited the resistance alleles of already mapped markers within the ROI and the other three inheriting the susceptible alleles. Thereafter, polymorphic markers were used to genotype identified recombinant individuals. PCR for polymorphism tests and genotyping as well as fragment analyses are as described earlier.

### Fire Blight Phenotypic Evaluations

We artificially inoculated interesting recombinant individuals and all four parents, ‘Idared,’ MAL0004, 07240–37 and ‘Golden Delicious’ with two strains of *E. amylovora* – Ea222 and Ea3049. In general, up to 10 replicates of each genotype were grafted onto M9 or M111 rootstocks, grown in the greenhouse, and subjected to inoculation. Greenhouse conditions were 25–27°C (day), 20°C (night), and 85% air humidity. Inoculations were performed in the quarantine greenhouse on plants with a minimum length of 25 cm by incising the two youngest leaves with a pair of scissors dipped into *E. amylovora* inoculum. Inoculum concentrations of 10^9^ cfu/ml were used for both strains (Emeriewen et al., 2017a). At 28 days post inoculation (dpi), we measured shoot length and lesion length (in cm) of replicates of each genotype, transformed the data into percentage lesion length (PLL) per shoot, and calculated the averages.

### Mapping and Genetic Analyses of the Region of Interest

We concentrated on the ROI for mapping. Marker data of individuals of the three populations were analyzed and mapped using JoinMap® 5.0 (Van Ooijen, 2018). Where it was not necessary to genotype non-recombinant individuals with newly developed markers, we deduced the genotypic data of these non-recombinant individuals. The mean and median PLL of recombinant individuals was calculated and individuals with PLL values lower than the median PLL were classed as resistant, whereas those with higher PLL were classed as susceptible. We transformed phenotypic data (PLL) into binary marker data for genetic mapping as described elsewhere (Emeriewen et al., 2018). We also deduced the phenotype of non-recombinant individuals of 17229 and 17230 populations using their genotypic data, since it was not necessary to phenotype non-recombinant individuals. For example, where an individual possessed the resistance alleles of all ROI markers, these individuals were deemed to possess the resistant phenotype. Similarly, where an individual possessed the susceptible alleles of all ROI markers, these individuals were deemed to possess the susceptible phenotype. MapChart (Voorrips, 2002) was used for the map comparisons.

**Figure 1:**
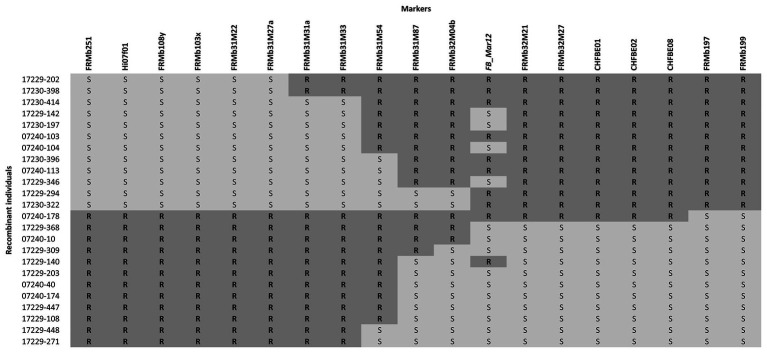
Graphical representation of the ROI with 24 informative recombinant individuals between markers FRMb31M27a and FRMb197 phenotyped with Ea222. *FB_Mar12* is the presumed position of the fire blight resistance gene. Phenotypic results of five recombinants did not fit the presumed locus position. R = resistant; S = susceptible.

For the establishment of the recombination table (Figure 1), 20% of genotypes surrounding the median PLL obtained by inoculation with Ea222 were deleted.

### Comparative Sequence Analyses of *FB_Mar12* and FB_E Markers on the *M. baccata* Draft Genome

Three markers (CHFBE01, CHFBE02, and CHFBE08), which are specific to and significantly linked to the fire blight resistance QTL/candidate genes of ‘Evereste’ on LG12 also map to MAL0004 ROI. Parravicini et al. (2011) reported that CHFBE02 co-segregates with FB_E, with CHFBE01 and CHFBE08 a few recombinations away upstream and downstream of FB_E, respectively. Sequences of markers flanking *FB_Mar12* and those reported by Parravicini et al. (2011), including candidate gene-specific primers, were BLAST-searched on the *M. baccata* draft genome (Chen et al., 2019) to ascertain their respective positions relative to both loci.

### Gene Prediction Analyses Using Scaffolds of the *M. baccata* Draft Genome

Following sequence analyses, the resultant scaffolds of *M. baccata*, where the putative *FB_Mar12* flanking markers and FB_E gene specific markers were positioned, were used to predict open reading frames (ORFs) using FGENESH with algorithms for dicot *Arabidopsis*. The predicted proteins were analyzed using the National Centre for Biotechnology (NCBI) Blastp program (Altschul et al., 1997) and ExPASy PROSITE (Bairoch, 1991; Hulo et al., 2008) to predict their domains, families, and functional sites.

## Results

### Increase of Mapping Individuals and Identification of Recombinants

Seven hundred and forty-seven (747) individuals from two new populations, 17229 and 17230, together with the original 145 individuals of 07240 population, bringing the total number of mapping individuals to 892, formed the basis of this study. Genotyping of these 747 individuals with closely linked markers in the ROI (interval between FRMb251 and FRMb199 containing *FB_Mar12*), resulted in the identification of 49 individuals showing recombination events between FRMb251 and FRMb199 (Table 1). Of these 49 individuals, 35 were from the 17229 population, whereas 14 were from the 17230 population. Thirteen individuals from the 07240 population also showed recombination events between FRMb251 and FRMb199, hence the total number of recombinant individuals between both markers bracketing the QTL was 62. These 62 individuals formed the basis of further genetic analyses.

### Marker Development and Identification of Significant Recombination Events Within the ROI

The corresponding ROI on GDDH13 spanned 2.3 megabases (Mb), i.e., from the region of 30,406,690–32,679,162, following BLAST-search of closely linked markers. Within this region on GDDH13, 40 primer pairs (Table 2) were developed enclosing SSR motifs. Of these, 20 were polymorphic in the mapping genotypes (Table 3). Since several of the polymorphic markers were located very close to each other in the ROI, only nine markers, which represent the entire region, were applied to the 62 recombinant individuals and subsequently mapped to avoid over clustering of markers in the ROI. Genotyping of the 62 recombinant individuals with these nine markers led to the reduction of the ROI between new marker FRMb31M27a and FRMb197 containing 29 recombinant individuals.

**Table 3:** Polymorphic markers and allele sizes.

Marker name	Allele sizes in base pairs (bp)			
	MAL0004	‘Idared’	‘GD’	07240–37
^†^FRMb31M22	184, Ø	192	188, 200	184, 192
FRMb31M23	202, 215	208	--	208, 215
^†^FRMb31M27a	211, Ø	198	224, 238	198, 211
^†^FRMb31M31a	Ø, 181	178	176, 178	178, Ø
^†^FRMb31M33	191, Ø	187	187	187, 191
FRMb31M35	Ø, 265	246	--	Ø, 246
FRMb31M36a	170, 174	172, 175	--	170, 175
FRMb31M48	Ø, 229	231	--	229, 231
FRMb31M52	139, 149	151	--	149, 151
^†^FRMb31M54	153, 161	150	163, 165	150, 161
FRMb31M61a	171, 190	180	--	190, 180
FRMb31M67	179, 183	239, 243	--	--
FRMb31M69	Ø, 196	213	207, 232	Ø, 213
FRMb31M7	217, 232	216	--	217, 216
FRMb31M80	208, 213, 232, 235, 241	225, 243	--	--
FRMb31M85	209, 213, 233, 236, 242	225, 243	--	--
^†^FRMb31M87	152, 170	144	150, 177	144, 170
^†^FRMb32M04b	194, 228	228	209, 242	194, 228
^†^FRMb32M21	167, 276	168	174	168, 276
^†^FRMb32M27	131, 160	--	150, 170	160

Allele sizes are according to Schuelke (2000); underlined are resistance alleles; Ø = null alleles ^†^markers, which were mapped to the ROI.

### Phenotypic Evaluations of Individuals

**Figure 2:**
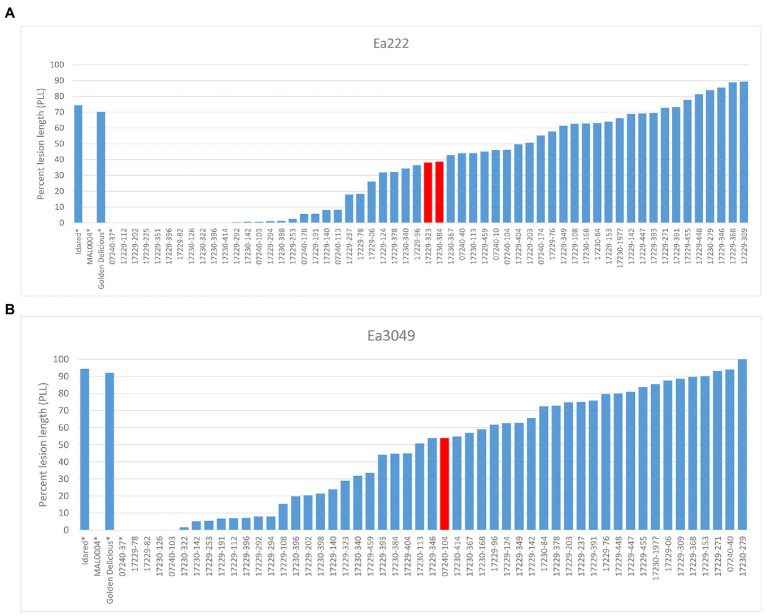
Results of phenotypic evaluation of parents and recombinant individuals for Ea222 (A), Ea3049 (B). The bars in red indicate the median PLL. ^*^parents.

We analyzed 58 recombinant individuals in total, including the 29 recombinants, which showed significant recombination events within the ROI enclosed by markers FRMb31M27a and FRMb197, with two strains of the bacterium: the moderately virulent Ea222 and the highly virulent Canadian strain, Ea3049. MAL0004, ‘Idared,’ 07240–37 and ‘Golden Delicious’ served as controls for artificial shoot inoculations. Results showed that whereas the resistant parents MAL0004 and 07240–37 showed no fire blight symptoms, recording an average PLL of 0% for both strains, the susceptible parents, ‘Idared’ and ‘Golden Delicious’ recorded over 70% necrosis for both strains (Figures 2A,B). Figures 2A,B also show the distributions of the resistance/susceptibility levels (i.e., fire blight severity) of the recombinant individuals. The mean PLL of the 57 recombinant individuals inoculated with Ea222 was 36.3, whereas for Ea3049 it was 47.6 for 50 recombinant individuals. In addition, there were 12 individuals, which recorded below 1% with Ea222, 10 of which recorded 0% (i.e., no disease symptom). In contrast, only three individuals recorded 0% with Ea3049. The median PLL values were 38.4 and 53.8 for Ea222 and Ea3049, respectively, with a correlation of 0.78. As expected, Ea3049 was more aggressive on the recombinant individuals than Ea222. There were only a few cases, where recombinant individuals recorded a higher severity for Ea222 than for Ea3049. Figure 1 shows the recombination table after removing five genotypes whose PLL were within the cut-off of 20% around the median PLL.

### Genetic Analyses and Mapping of the ROI

**Figure 3:**
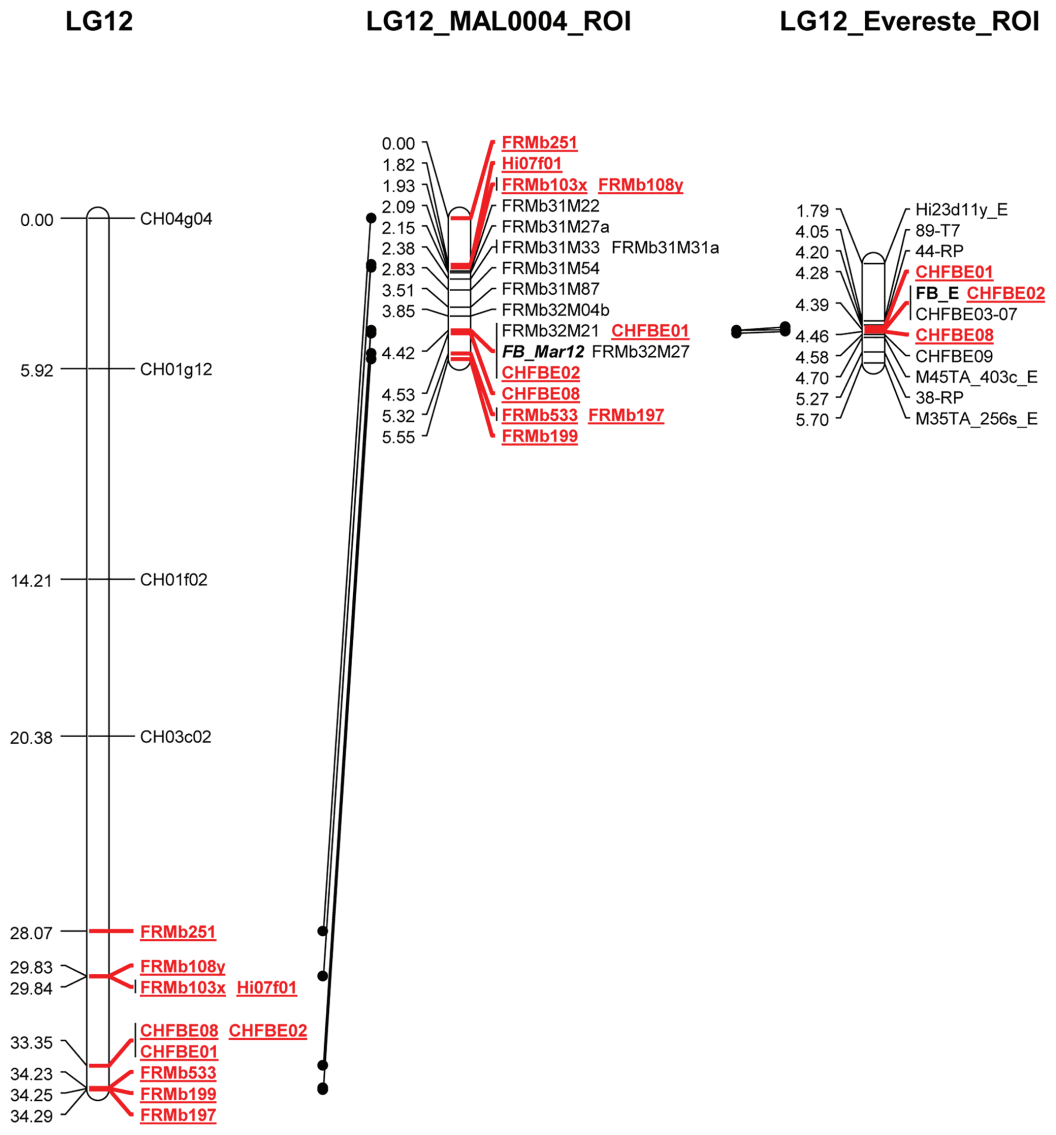
Genetic maps of *Malus* ×*arnoldiana* LG12 (Emeriewen et al., 2017a) and the region of interest (LG12_MAL0004_ROI) on LG12 containing *FB_Mar12*, in comparison to the ‘Evereste’ fire blight resistance locus (LG12_Evereste_ROI). Markers in common are highlighted in red and underlined.

We mapped 20 markers to the ROI including *FB_Mar12* as a phenotypic marker. This represents 10 additional markers than was previously reported for the ROI (Emeriewen et al., 2017a). For genetic mapping of the ROI, we deduced the genotypic data of non-recombinant individuals for the nine markers applied on only the recombinants in order to facilitate genetic mapping of the ROI with the entire 892 mapping individuals. To integrate *FB_Mar12* as a binary marker, we transformed phenotypic data (PLL) of recombinant individuals as well as the 07240 individuals (data from Emeriewen et al., 2017a) – into binary marker data. Therefore, we deduced the phenotypes of non-recombinant individuals by assigning 0 (i.e., resistant phenotype) and 1 (susceptible phenotype) to individuals possessing resistant and susceptible alleles of tightly linked markers, respectively. This process facilitated the delimiting of the region containing the locus from 6.22 (Emeriewen et al., 2017a) to 0.57 cM (Figure 3). Two new markers FRMb32M21 and FRMb32M27 together with CHFBE01 and CHFBE02 co-segregated with *FB_Mar12*. The marker recombinations calculated by JoinMap® 5.0 (Van Ooijen, 2018) for the ROI (Figure 3) is in agreement with the marker data for the 24 informative recombinant individuals shown in Figure 1. However, the phenotype of five individuals did not correspond with their genotype.

### Comparative Analyses of *FB_Mar12* and FB_E Regions on the *M. baccata* Draft Genome

We used the sequences of markers within the ROI (Table 4) as query sequences to perform BLAST searches on the draft genome of *M. baccata* – a wild *Malus* species being one parent of the hybrid *M. ×arnoldiana*. Markers CHFBE01, CHFBE02, and CHFBE08 are strongly linked to FB_E – the fire blight resistance locus of the ornamental cultivar ‘Evereste,’ with CHFBE02 co-segregating with FB_E (Parravicini et al., 2011). Following sequence analyses, we located the fragments of all three FB_E markers on *M. baccata* scaffold785 (Table 4). Interestingly, six of eight ORF-specific primers proposed by Parravicini et al. (2011) aligned to scaffold785 (Table 4). The markers FRMb32M21 and FRMb32M27, which also co-segregate with *FB_Mar12*, both aligned to *M. baccata* scaffold778 and scaffold1226/746, respectively. Marker FRMb32M04b, which defines the upstream border of the 0.57 cM interval containing *FB_Mar12* cluster in our map, aligned to *M. baccata* scaffold616. We used the sequences of these five scaffolds for gene prediction analyses.

**Table 4:** Markers from LG12 fire blight resistance regions of *M. ×arnoldiana* and ‘Evereste’ and their location on the *M. baccata* draft genome.

Marker	Source	*M. baccata* scaffold located
FRMb31M87	This study	Scaffold616
FRMb32M04b	This study	Scaffold616
FRMb32M21	This study	Scaffold778
FRMb32M27	This study	Scaffold1226; Scaffold746
CHFBE01	Parravicini et al., 2011	Scaffold785
CHFBE02	Parravicini et al., 2011	Scaffold785
CHFBE08	Parravicini et al., 2011	Scaffold785
MdE-EaK1^†^	Parravicini et al., 2011	Scaffold785
MdE-EaK2^†^	Parravicini et al., 2011	Scaffold785
MdE-EaK3^†^	Parravicini et al., 2011	Scaffold785
MdE-EaK4^†^	Parravicini et al., 2011	Scaffold1506; Scaffold399
MdE-EaKN^†^	Parravicini et al., 2011	Scaffold785
MdE-EaK5^†^	Parravicini et al., 2011	Scaffold785
MdE-EaK6^†^	Parravicini et al., 2011	Scaffold785
MdE-EaK7^†^	Parravicini et al., 2011	--

† Markers, which were developed from open reading frames (ORFs).

Gene prediction analyses using FGENESH with algorithms for *Arabidopsis* resulted in the prediction of several genes for each scaffold. Table 5 lists the ORFs with plant disease-related domains for each scaffold. The scaffolds potentially harboring *FB_Mar12* possessed genes with domains distinct to plant disease resistance, i.e., serine/threonine kinase and LRR domains. Ten serine/threonine kinase genes were predicted on scaffold785 containing the FB_E candidate genes, for which Parravicini et al. (2011) proposed similar kind of genes. Of the 10 serine/threonine kinase genes, five were homologs of the ‘Evereste’ ORFs (Table 5). ORFs with leucine rich repeats, serine/threonine kinase domains, and toll/interleukin receptor domains were predicted on scaffold616. The least number of ORFs were predicted on scaffold778, scaffold1226, and scaffold746.

**Table 5:** ORFs with plant disease-related domains predicted on the *M. baccata* scaffolds.

Scaffold	ORFs	aa length	Domain(s)	Exons
Scaffold616	FGenesh1	682	TIR	6
	FGenesh10	889	TIR	7
	FGenesh12	869	TIR-LRR	6
	FGenesh31	1701	Protein_Kinase-Integrase	6
	FGenesh40	713	Protein_Kinase	5
	FGenesh41	582	Protein_Kinase	3
	FGenesh42	634	Protein_Kinase	11
	FGenesh43	1,176	Protein_Kinase	14
				
Scaffold778	FGenesh2	594	Protein_Kinase	5
	FGenesh4	530	Protein_Kinase	7
	FGenesh34	1,053	Protein_Kinase	3
				
Scaffold1226	FGenesh20	440	Protein_Kinase	7
				
Scaffold746	FGenesh38	341	Protein_Kinase	6
	FGenesh40	326	Protein_Kinase	5
				
Scaffold785	FGenesh8	582	Protein_Kinase	5
	FGenesh20^†^	388	Protein_Kinase	1
	FGenesh21	354	Protein_Kinase	1
	FGenesh23^†^	558	Protein_Kinase	3
	FGenesh29^†^	412	Protein_Kinase	1
	FGenesh32^†^	403	Protein_Kinase	1
	FGenesh35	363	Protein_Kinase	2
	FGenesh38^†^	400	Protein_Kinase	1
	FGenesh39	492	Protein_Kinase	4
	FGenesh41	401	Protein_Kinase	1
	FGenesh71	729	LRR	1

ORF: open reading frames; aa: amino acid; Protein_Kinase: serine/threonine kinase signature; LRR: Leucine rich repeats; TIR: Toll/Interleukin 1 receptor.

† Homologs of candidate genes predicted by Parravicini et al. (2011).

## Discussion

There is a variability of fire blight resistance in *Malus* with wild *Malus* accessions/species possessing stronger resistance to *E. amylovora* (Emeriewen et al., 2019). Fire blight resistance loci of three wild *Malus* species, *M. floribunda* 821 (Mf821), ‘Evereste’ and *M. ×arnoldiana* MAL0004 are located at the distal end of LG12 (Durel et al., 2009; Emeriewen et al., 2017a). Of these three resistance loci, only the locus of ‘Evereste’ has been fine mapped, leading to the identification of candidate genes (Parravicini et al., 2011). Here, we report the fine mapping of the fire blight resistance locus of the highly resistant *M. ×arnoldiana* accession MAL0004. *FB_Mar12* was initially detected using a population size of 145 individuals following artificial inoculation with two strains of *E. amylovora*, Ea222 and Ea3049 (Emeriewen et al., 2017a). The significant increase of the mapping individuals, and the development and mapping of nine closely linked markers facilitated the identification of important recombinant individuals within the ROI. Although we used 892 individuals for this study, much less than the 2,703 reported for the fine mapping of the ‘Evereste’ fire blight region (Parravicini et al., 2011), we detected 29 highly significant recombinant individuals in a 2.9 cM interval within the *FB_Mar12* ROI. This interval is comparatively larger than the 0.5 cM in ‘Evereste,’ where Parravicini et al. (2011) identified 15 recombinant individuals within the FB_E region. In other fire blight resistance loci fine mapping studies, Fahrentrapp et al. (2013) and Emeriewen et al. (2018) identified 33 and 31 recombinant individuals in 1.53 and 1.2 cM intervals, respectively, from which the fire blight candidate genes of Mr5 – *FB_MR5* and *M. fusca* – *FB_Mfu10* were predicted. The phenotypes of the 29 recombinants enabled the precise mapping of *FB_Mar12* to a 0.57 cM interval, thus delimiting the region significantly from 6.22 cM reported previously (Emeriewen et al., 2017a). However, five recombinant individuals within this region showed phenotype-genotype incongruity. The reason for this phenomenon remains unclear, however, mislabeling of individuals prior to phenotyping when dealing with such large population size is a possible reason. Unexplainable genotype-phenotype incongruities were observed also in the fire blight resistance regions of Mr5 (Fahrentrapp et al., 2013), *M. fusca* (Emeriewen et al., 2018), and ‘Evereste’ (Parravicini et al., 2011). Furthermore, our mapping results suggest that the fire blight resistance loci of *M. ×arnoldiana* and ‘Evereste’ may be closely related, if not the same (Figure 3). The regions containing both loci share three markers, CHFBE01, CHFBE02, and CHFBE08. Parravicini et al. (2011) reported that CHFBE02 co-segregated with the FB_E with CHFBE01 and CHFBE08 mapping at 0.11 and 0.07 cM upstream and downstream, respectively. Here, *FB_Mar12* co-segregates with both CHFBE01 and CHFBE02 with CHFBE08 0.11 cM downstream. Analyses of the FB_E region with the nine markers developed in this study will confirm if there are chromosomal rearrangements.

The availability of the *M. baccata* draft genome (Chen et al., 2019) presented an excellent opportunity to analyze the fire blight resistance genomic regions of ‘Evereste’ and *M. ×arnoldiana* using the genome of this wild *Malus* species, since *M. ×arnoldiana* is a hybrid species of *M. baccata × M. floribunda* (Fiala, 1994), with many accessions reported as moderately or highly resistant to fire blight (den Boer and Green, 1995; Luby et al., 2002; Dougherty et al., 2021), unlike ‘Golden Delicious,’ which is highly susceptible to the disease. Comparative analyses of FB_E and *FB_Mar12* marker sequences on the *M. baccata* draft genome (Chen et al., 2019) shed more light into the similarities of the fire blight loci of ‘Evereste’ and *M. ×arnoldiana* (Table 4). It is significant that the fragments of the FB_E markers as well as markers designed from ORFs, which were predicted from the BAC (bacterial artificial chromosome) clone spanning the ‘Evereste’ fire blight resistance region (Parravicini et al., 2011) aligned to *M. baccata* scaffold785. Thus, it is not surprising that the ORFs predicted using the sequence of Scaffold785 resulted in homologs and genes with similar domains as the fire blight candidate genes predicted by Parravicini et al. (2011), i.e., serine/threonine kinase domains and leucine rich receptor domains. Although we did not develop a BAC library for genome walking approach in the present study, we postulate that the ORFs predicted on the homologous region of the *M. baccata* draft genome are indeed putative fire blight resistance genes of *M. ×arnoldiana*. In fact, the distal end of LG12 appears to contain an abundance of putative resistance genes. The fact that similar genes were predicted on scaffolds containing markers that surround the putative *FB_Mar12* region in the *M. baccata* genome is a strong indication of the similarity of the regions. It is therefore highly probable that genome-walking approach with BACs to determine the physical regions of *M. ×arnoldiana* and ‘Evereste’ (Durel et al., 2009) fire blight resistance regions will result in the detection of these genes.

The prediction of several putative candidate genes within the *FB_Mar12* and FB_E regions is in contrast to the fire blight resistance regions of Mr5 (Fahrentrapp et al., 2013) and *M. fusca* (Emeriewen et al., 2018), where single candidate genes were predicted. Indeed, this could pose a challenge in cloning studies to prove the functionality of the genes as it will be difficult to pinpoint the most probable candidate gene accurately. Another possible question for future research is whether two or more of these genes are acting together to provide fire blight resistance. The potential difference in resistance mechanisms between MAL0004 and ‘Evereste’ should be taken into account even though their respective resistance loci on LG12 appear to be, at least closely related, with similar genes of interest. Eop1 mutant strain of *E. amylovora* caused considerable disease symptoms in ‘Evereste’ but not in MAL0004 (Wöhner et al., 2018). The potential difference of resistance mechanisms between MAL0004 and ‘Evereste’ might be attributed to the fact that MAL0004 has another fire blight resistant wild species – *M. baccata* as a parent. The pedigree of ‘Evereste’ contains apple cultivars ‘Red Delicious,’ ‘Rome Beauty,’ and ‘Jonathan’ as well as Mf821 (Durel et al., 2009). Genetic analyses of their respective progeny with this mutant strain should confirm the difference in resistance mechanisms of both donors and otherwise (Emeriewen et al., 2019). If indeed, Eop1 breaks down the fire blight resistance of ‘Evereste’ and not MAL0004, then Eop1 would likely break down the major determining factor underlying FB_E, but not *FB_Mar12*. This premise is predicated on the results of Mr5 fire blight resistance (Peil et al., 2011; Vogt et al., 2013; Wöhner et al., 2014). The overexpression of *FB_MR5* in transgenic ‘Gala’ provided resistance to Mr5 avirulent strains but not to the Mr5-virulent strain ZYRKD3-1 (Broggini et al., 2014). ZYRKD3-1 is an avrRpt2_EA_ deletion mutant – an analog of the effector protein avrRpt2 of *Pseudomonas syringae* (Zhao et al., 2006), which is responsible for the breakdown of the fire blight resistance of Mr5, leading to the first known gene-for-gene interaction between *Malus* host and *E. amylovora* (Vogt et al., 2013; Emeriewen et al., 2019). The results of Eop1 causing fire blight on *M. floribunda* 821 and ‘Evereste’ is indicative of another gene-for-gene interaction (Wöhner et al., 2018).

## Concluding Remarks and Future Work

Taking the strain specificity of fire blight resistance into account, breeding to achieve durable resistance to various strains of the pathogen would ultimately require the pyramiding of differently acting genes. *FB_Mar12* (Emeriewen et al., 2017a), which maps to the distal part of LG12, is a strong fire blight resistance candidate, given the potential difference in fire blight resistance mechanism from two other loci on LG12 – E12 and Mf12 (Durel et al., 2009). The works presented here are the first steps toward uncovering the major determining factor underlying *FB_Mar12*. The putative candidate genes predicted using the *M. baccata* draft genome (Chen et al., 2019) would have to be confirmed prior to complementing studies. The conventional approach of characterizing the physical region containing fire blight resistance loci (Parravicini et al., 2011; Fahrentrapp et al., 2013; Emeriewen et al., 2018) may not be necessary as resequencing of the *FB_Mar12* region by targeted sequencing of DNA molecules (Madsen et al., 2020) or by employing CRISPR-Cas9 target enrichment and Nanopore sequencing (López-Girona et al., 2020) to resolve the *FB_Mar12* region would be perhaps better approaches than genome-walking. The functional analyses of candidate genes of *M. ×arnoldiana* and ‘Evereste’ will advance our understanding of mechanisms underlying LG12 fire blight resistance loci. In conclusion, the development of a dense genetic map for *M. ×arnoldiana*, which represents the 17 linkage groups of *Malus*, should facilitate for the detection of putative additional loci contributing to resistance, if such loci are indeed present in *M. ×arnoldiana* genome.

## Data Availability Statement

The raw data supporting the conclusions of this article will be made available by the authors, without undue reservation.

## Author Contributions

All authors conceptualized the study. OFE performed marker analyses, data analyses, mapping, and drafted the manuscript. AP established the populations, analyzed the marker data, analyzed the phenotypic data and mapping, and supervised the project. KR performed the phenotyping and data analyses. HF provided the platform and intellectual discussions. MM contributed to intellectual discussions and is project partner. All authors read, edited, and approved the final version of the manuscript.

### Conflict of Interest

The authors declare that the research was conducted in the absence of any commercial or financial relationships that could be construed as a potential conflict of interest.
